# Diffusion Mechanism Modeling of Metformin in Human Organic Cationic Amino Acid Transporter one and Functional Impact of S189L, R206C, and G401S Mutation

**DOI:** 10.3389/fphar.2020.587590

**Published:** 2021-02-09

**Authors:** Leydy Cano, Alejandro Soto-Ospina, Pedronel Araque, Maria Antonieta Caro-Gomez, Maria Victoria Parra-Marin, Gabriel Bedoya, Constanza Duque

**Affiliations:** ^1^ Universidad de Antioquia, Medellín, Colombia; ^2^ Universidad EIA, Envigado, Colombia; ^3^ Cecoltec, Medellin, Colombia; ^4^ Tecnológico de Antioquia, Medellín, Colombia; ^5^ Universidad Cooperativa de Colombia, Medellín, Colombia

**Keywords:** pharmacogenetics, type 2 diabetes, metformin, structural biology, transport mechanism

## Abstract

Metformin used as a first-line drug to treat Type 2 Diabetes Mellitus is transported via organic cation channels to soft tissues. Mutations in the SLC22A1 gene, such as Gly401Ser, Ser189Leu, and Arg206Cys, may affect the drug’s therapeutic effect on these patients. This study aims at proposing a potential structural model for drug interactions with the hOCT1 transporter, as well as the impact of these mutations at both topological and electronic structure levels on the channel’s surface, from a chemical point of view with, in addition to exploring the frequency distribution. To chemically understand metformin diffusion, we used an open model from the protein model database, with ID PM0080367, viewed through UCSF Chimera. The effect of the mutations was assessed using computational hybrid Quantum Mechanics/Molecular Mechanics, based on the Austin Model 1 semi-empirical method using Spartan 18’ software. The results demonstrate coupling energy for metformin with amino acids F, W, H and Y, because of the interaction between the metformin dication and the electron cloud of π orbitals. The mutations analyzed showed changes in the chemical polarity and topology of the structure. The proposed diffusion model is a possible approach to the interaction mechanism between metformin and its transporter, as well as the impacts of variants, suggesting structural changes in the action of the drug. Metformin efficacy considerably varies from one patient to another; this may be largely attributed to the presence of mutations on the SLC22A1 gene. This study aims at proposing a potential structural model for metformin-hOCT1 (SLC22A1) transporter interaction, as well as the identification of the effect of mutations G401S (rs34130495), S189L (rs34104736), and R206C (616C > T) of the SLC22A1 gene at the topological and electronic structure levels on the channel surfaces, from a chemical viewpoint. Our results demonstrated that the coupling energies for metformin with aromatic amino acids F, W, H and Y, because of the interaction between the metformin dication and the electron cloud of π orbitals. Changes in the chemical environment’s polarity and the structure’s topology were reported in the mutations assessed. The diffusion model proposed is a potential approach for the mechanism of interaction of metformin with its transporter and the effects of variants on the efficacy of the drug in the treatment of type 2 diabetes. The assessment of the frequency of these mutations in a sample of Colombian type 2 diabetes patients suggests that different SLC22A1 gene variants might be involved in reduced OCT1 activity in the Colombian population since none of these mutations were detected.

## Introduction

Metformin is mostly present as a hydrophilic cation at a physiological pH (Liang and Giacomini, 2017). It is a biguanide used as a first-line drug to treat Type 2 Diabetes (T2DM) (Markowicz-Piasecka et al., 2016; Sanchez-Rangel and Inzucchi, 2017) because it helps decrease blood glucose levels by 33%. Unlike other drugs, metformin reduces triacylglycerol levels in the blood by 10–15% and cuts the risk of heart attack or stroke by ≈ 50%. Overall, by itself, metformin is highly effective and one of the few oral agents used to treat T2DM that promotes weight loss; therefore, it is a good drug for treating and/or preventing T2DM (Markowicz-Piasecka et al., 2016; Florez, 2017). Although metformin’s mechanism of action is unclear, evidence suggests that this biguanide improves hyperglycemia by decreasing intestinal glucose absorption, peripheral insulin sensitivity, and inhibits of hepatic gluconeogenesis and glycogenolysis (Wang et al., 2002; Shu et al., 2007; Nies et al., 2011; Markowicz-Piasecka et al., 2016; Foretz and Guigas, 2019).

Despite its widespread use, there are substantial variations in its response among individuals, with ∼35% of patients fail to achieve initial glycemic control (Pascale et al., 2018). This is because of the differences in drug availability and action, attributed to polymorphisms in the genes that regulate metformin pharmacokinetics and pharmacodynamics (Mahrooz et al., 2015; Florez, 2017; Pascale et al., 2018).

Metformin’s entry into and exit from target tissues are mediated by organic cation transporters (OCT), belonging to the solute carrier 22 family (SLC22) of membrane proteins that play a crucial role in the drug’s absorption, distribution, and renal excretion (Markowicz-Piasecka et al., 2016; Pascale et al., 2018; Santoro et al., 2018).

Metformin’s binding and transport mechanism has not been completely determined, mostly because of the absence of any trace of a crystal structure. Membrane proteins, such as OCT, participate in almost all cell activities. Therefore, defining its 3D structure would help to explain its biological function (Lin et al., 2015).

The known crystal structures from transmembrane proteins have been used to predict the 3D structures of other proteins sharing common structural properties for a long time. OCT2 structure in rats was predicted using the crystal structure of LacY permease (*Escherichia coli*) (Abramson et al., 2003; Zhang et al., 2005) and the oxalate transporter of *Oxalobacter formigenes* (Ubarretxena-Belandia et al., 2003) as a template. For OCT1, there is a structural model, recorded in the protein model database (PMD) with ID PM0080367, developed by Dakal et al., 2017 using structure prediction techniques, which facilitated the modeling and prediction of tertiary structures of proteins such as OCT1 transporters (Dakal et al., 2017) through theoretical structural biology approaches. Other studies used computer simulation techniques, such as molecular docking, to predict the affinity of OCT transporters to their tRet molecules and visually examine the impact of genetic variants on metformin transport, as proposed by Green (Green, 2015). Nevertheless, these methods fail to provide accurate predictions of the effects of mutations on the pharmacodynamics of antidiabetic treatments (Schlessinger et al., 2013; Chen et al., 2017).

In humans, the OCT1 transporter is codified by the SLC22A1 gene, located on chromosome 6q26, composed of 11 exons and comprising ∼37 kb (Wang et al., 2002). This gene is highly polymorphic and certain variants have an effect on the transporter’s function in ethnically diverse populations (Mahrooz et al., 2015; Mato et al., 2018). Previously, studies reported that people with reduced-activity variants of this gene may exhibit a decrease in the amount of metformin available in the liver and a reduced therapeutic response (Shu et al., 2007; Yang et al., 2014).

Giacomini et al. pioneered several of these studies and showed that the presence of at least one of the four reduced-function variants (R61C, G401S/rs34130495, M420/rs72552763 and/or G465R/rs34059508) mitigates metformin’s glycemic effects (Shu et al., 2007), probably because an alteration in intracellular metformin transport, thus resulting in higher peak plasma concentrations and area under the curve (AUC) of the drug (Shu et al., 2008). Furthermore, a study on the secondary effects of metformin on 251 metformin-intolerant and 1951 tolerant individuals by Genetics of Diabetes and Audit Research Tayside Study (GoDARTS) researchers in Tayside, Scotland, demonstrated that the presence of two additional reduced-function alleles of G401S increases the likelihood of metformin intolerance, thus inducing the accumulation of metformin in enterocytes (Dujic et al., 2015). Another variant associated with decrease in metformin absorption is S189L (rs34104736) (Mato et al., 2018). Both M420 and S189L have been primarily identified among populations of the European ancestry (Song et al., 2008; Chen et al., 2010). However, Chen et al. (2010) while the non-synonymous R206C variant, although rare among Chinese and Japanese populations, contributes to an altered metformin response in individuals carrying the mutation by reducing significantly OCT1 expression and function (Chen et al., 2010).

The OCT-family proteins are a potential therapeutic target. Therefore, identifying the mechanism by which metformin interacts with this transporter is crucial because the information obtained may contribute to advances in personalized medicine. In recent years, studies have focused on understanding the effect of genetic polymorphisms in the pharmacokinetic modulation of metformin transporters. However, the molecular mechanism of interaction between the drug and transporter is still unclear; therefore, this study proposes a potential structural model for the diffusion and interaction of this drug with the hOCT1 transporter, as well as the identification of the effect G401S, S189L, and R206C at the topological and electron structure levels, as well as explore the presence of these rare functional variants in a sample of T2DM patients from Colombia.

## Methodology

### hOCT1-Mediated Metformin Transport Modeling

#### Characterization of α Fractions of Metformin

To identify the different pH-dependent structural forms, the percentage of microspecies was calculated for metformin, for a physiological pH scale, using the Chemicalize software and the data produced is complemented with the information from the literature for the alpha fractions of metformin (Atlassian Confluence; Ciarimboli et al., 2015).

#### Metformin Geometry Optimization

The structural model for metformin was developed using Spartan 18’ software (Wavefunction) purchased license (Wavefunction, 1991). The molecular optimization was conducted following minimum geometric and global energy parameters using the Hartree–Föck method with a 3–21G* basis set in water, considering a solvation shell [SM8].

#### Metformin Density Surfaces and Electrostatic Potential Maps

The density and electrostatic potential map were calculated using the Spartan 18’ software surface tool (Wavefunction, 1991). The computation work generated an output file with an illustrative image of the density surface based on the load distribution and electrostatic potential map for an energy sweep in a range between −200 and 200 kJ/mol.

#### Characterization of the hOCT1 Channel in Databases

Initially, a search was performed in the RCSB Protein Data Bank databases for the hOCT1 organic cation transporter; however, no crystallized structure could be identified by experimental techniques such as X-ray diffraction, cryogenic electron microscopy, or solid-state nuclear magnetic resonance. After conducting a comprehensive literature search, channel modeling was conducted using I-TASSER using the GLUT3 template with PDB ID:5C65 (Quality, 2007; Zhang, 2008; Yang et al., 2015; Dakal et al., 2017). The hypothetical model was validated and refined using stereochemistry and energy minimization. The results for the seven organic cation transporters predicted were published in the Protein Model database (http://srv00.recas.ba.infn.it/PMDB/main.php), and assigned the code PM0080367, followed by an assessment using the SAVES server, which was specifically downloaded for this study.

#### hOCT1 Hydropathy Index

The chemical environment was characterized using polarity by comparing metformin diffusion with respect to hOCT1 under its native structure and the variants that alter diffusion of the drug. To understand this interaction, Protscale (Swiss ExPASY suite) was implemented to calculate the modifications of the Kyte and Doolittle coefficients in proteins, which are related to the description of the native and mutant protein environments (Artimo et al., 2012). For this procedure, the primary sequence in FASTA format was used; the hydropathy index was then estimated with Kyte and Doolittle coefficients, commonly used for this type of calculation. This software allocates a score for each amino acid of hOCT1, within a range of integers considering both positive and negative signs. The amino acid is considered to be hydrophobic, if positive, and hydrophilic, if negative, with a threshold value of zero (Gasteiger et al., 2005).

#### Metformin and hOCT1 Structural Visualizers

To achieve a 3D idea of the system and understand the spatial distribution of the diffusion model in the hOCT1 channel, Deepview/Swiss-PdbViewer, version 3.7 (Guex and Peitsch, 1997) was used to facilitate the preliminary approach of the diffusion path based on the channel’s nature. The structures were subsequently visualized using UCSF Chimera v1.11 (Pettersen et al., 2004) because it allows the model to be configured as an atomic display with density belts or surfaces.

### Topological and Functional Effect of the hOCT1 Organic Cation Transporter for S189L, R206C, and G401S Variants

#### Energy Optimization of hOCT1 Mutations

S189L (rs34104736), G401S (rs34130495), and R206C (616C > T) mutations, reported in the literature, were selected because of their functional effect on the decrease in metformin uptake (Takane, 2008; Chen et al., 2010; Yoon et al., 2013; Mato et al., 2018). After selecting the variants, their effect was evaluated from a chemical viewpoint using Spartan14’ (Wavefunction, 1991). The Z-matrix was selected for regions close to the mutation and potential interaction areas, whereas geometric optimization was calculated based on a semi-empirical method considering the macromolecular system and the effects relating to atomic variants in the electronic structure. The Austin model1 (AM1) base was used with 10,000 iteration cycles. Considering this computational methodology, the bond length, angle changes, and measurement of the dihedral angles were calculated, in addition to the areas or volumes of ionization potential surfaces.

#### Density Surface and Electrostatic Potential Maps

The mutations assessed have different characteristics. To understand the changes in the electronic structure and anchoring ability, or lack thereof, the density and electrostatic potential map calculation were applied, using the Spartan 18’ software surface tool (Wavefunction, 1991). This tool was used to measure total surface areas for the potentials in Å^2^ and surface volumes in Å^3^ for the relevant regions.

#### Structural Visualizers

Deepview/Swiss-PdbViewer software was used to obtain a 3D idea of the system and understand the spatial distribution of the mutations assessed, as well as how they may affect metformin translocation or the structural changes that hinder interaction with metformin in the hOCT1 channel (Guex and Peitsch, 1997). The interaction lines of the hydrogen bonds for the structure of mutated hOCT1 channels were calculated and images were gathered and visualized using UCSF Chimera (Pettersen et al., 2004) because it allows the configuration of the structural model in belts, atoms, tangent spheres, or density surfaces.

#### Hydropathy Index of the Effect of the Variants on the hOCT1 Organic Cation Transporter

The behavior of the channel in terms of polarity is crucial toward understanding the effects of these variants with regard to the development of pathologies, because it can affect the net increase or decrease of anchor points that change the structure or spatial distribution (Cardona et al., 2018; Cardona-Pemberthy et al., 2018). Protscale, was used to calculate the modifications in proteins and the Kyte and Doolittle coefficients, related to the description of the native and mutant protein environments (Artimo et al., 2012). The primary sequence of the organic cation channel was used with a score assigned for each amino acid comprising the channel and which, in function of the size and sign of the value, may represent hydrophobic or hydrophilic interactions, thus confirming many of these changes based on the effect of translocation of the system or diffusion and affinity between the hOCT1 channel and metformin (Gasteiger et al., 2005).

### Frequency of Functional Variants on the SLC22A1 Gene in a Colombian Population

#### Colombian Study Population

Three hundred and ninety-three patients who had been diagnosed with T2DM were included in the study, 294 of which were treated with metformin and 99 treated with other drugs. These participants were part of the studies previously conducted at the Molecular Genetics Laboratory (GENMOL) at the University of Antioquia (Duque, 2011; Parra, 2012; Caro, 2018). The participants provided information regarding their socio-demographic data, personal history of metabolic syndrome diseases, lifestyle (such as diet and physical exercise), other medication anthropometric measurements (such as height, weight, and body mass index), waist circumference, hip circumference and waist-to-hip ratio, and biochemical values (such as glycosylated hemoglobin, blood glucose, total cholesterol, triglycerides, and high-density lipoprotein). This information was obtained from the studies conducted by Fernandez (2007), Duque (2011), Parra (2012), and Caro (2018), and approved by the Bioethics Committee of the Cooperative University of Colombia (Medellín-Antioquia), Approval no. 800-053 of 2014.

#### Genotyping

The non-synonymous variants G401S (rs34130495), S189L (rs34104736), and R206C (616C > T) on the SLC22A1 gene were genotyped by LGC Genomics Ltd. (Beverly, United States) using KASP-PCR (Kompetitive Allele Specific PCR).

## Results

### Metformin Characterization with Software Chemicalize

In this first part, the distribution of metformin microspecies was characterized with respect to physiological pH in different environments (such as saliva, stomach, small intestine, and blood) based on alpha fractions (Supplementary Figure S1). The microspecies’ values for the abovementioned biological pHs are listed in Table 1. Consequently, dicationic microspecies 4 was reported to be more predominant (100%) for pHs ranging from 1 to 8, considering the entire pH range within the human soma.

**TABLE 1:** Biological environment for metformin and percentage for each microspecies.

Physiological pH	% Microspecies1 (M.S1)	% Microspecies2 (M.S2)	% Microspecies3 (M.S3)	% Microspecies4 (M.S4)
Saliva pH:5.6	0.0	0.0	0.0	100.0
Stomach pH:2.0	0.0	0.0	0.0	100.0
Small intestine pH: 5.0	0.0	0.0	0.0	100.0
Blood pH: 7.0	0.0	0.0	0.0	100.0

After identifying the predominant microspecies that best represented metformin in a biological environment, a possible passive diffusion path was studied and proposed in which tyrosine, phenylalanine, tryptophan, and histidine were optimized as a possible route of interaction between the metformin dication and the OCT1 channel under a π-stacking interaction because of the aromaticity of the side chain.

### Proposal for the hOCT1 Organic Cation Transporter Model

**FIGURE 1:**
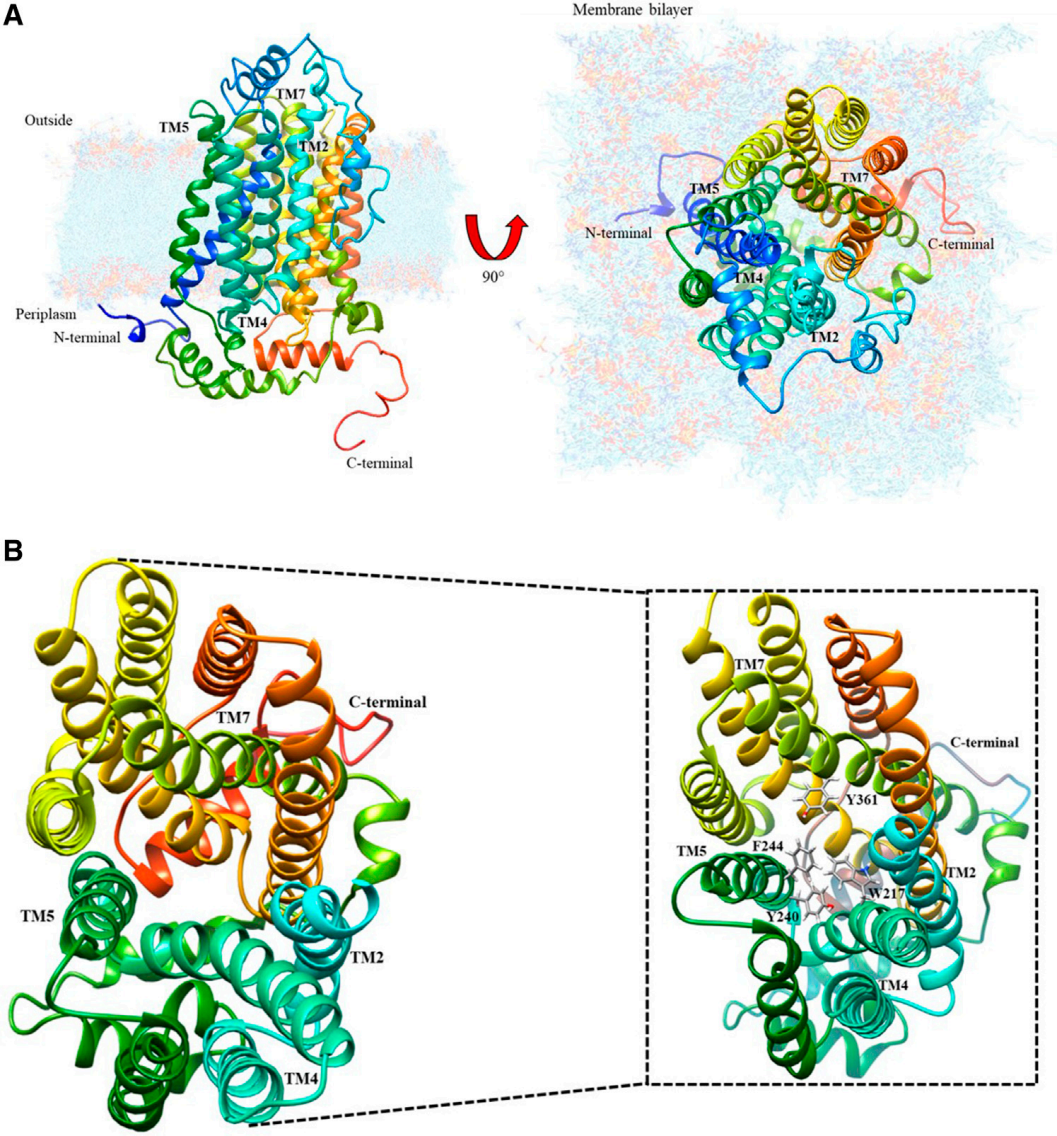
Organic cation transporter hOCT1: **(A)** Modeled by structure predictors with lateral and top-plane vision; **(B)** Top-view of the aromatic amino acid pocket; the magnified image identifies the aromatic amino acids present in the cation diffusion channel.

The protein model database (Protein data bank) search currently shows no experimentally obtained crystallized structure for the cation transporter. However, it has been modeled using homology modeling based on structure predictors, which was refined and validated with energy and stereochemical tools by Dakal et al. (2017). As shown in Figure 1A, this model was downloaded from the protein model database with ID PM0080367.

### Characterization of the Diffusion Path in hOCT1

After analyzing this model using the UCSF Chimera visualizer according to the bioinformatic characterization for the secondary structure (Dakal et al., 2017), there is an extracellular loop that has potential sites for N-glycosylation because of asparagines Asn71, Asn96, and Asn112. These are conformational changes that may generate the extracellular loop in the channel, thus inducing possible spatial transformations of TMH2, TMH4, TMH5, and TMH7, which allow metformin entry. Figure 1B shows the interior of the organic cation channel, formed by aromatic amino acids such as F, W, H and Y, which can intervene in metformin dication’s diffusion. The following is the visualization of the channel and the proposed passive diffusion path of interaction.

### Topological Localization of hOCT1 in the Lipid Membrane Based on the Hydropathy Index

**FIGURE 2:**
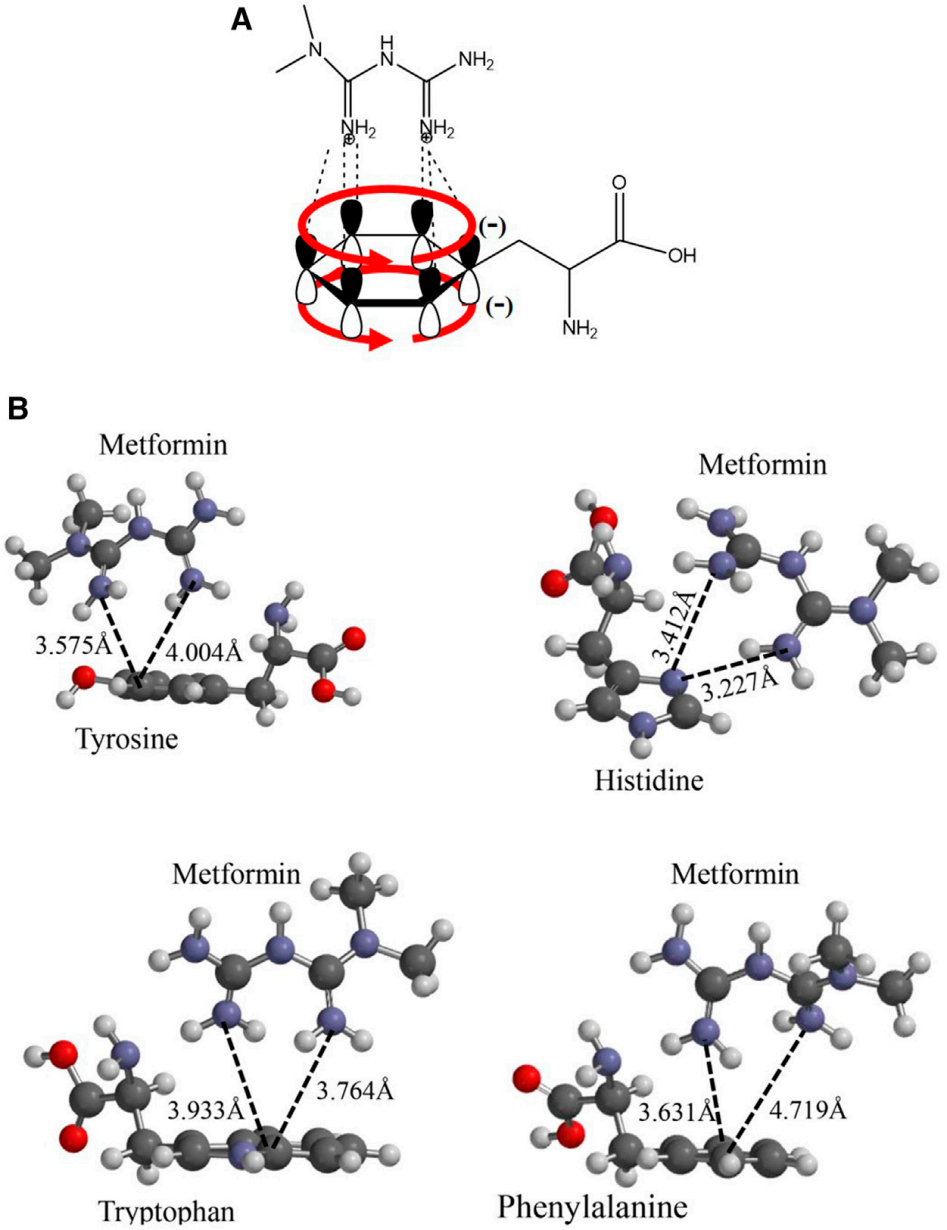
Proposed interaction between the metformin cation and the amino acids p orbitals with aromatic functional groups that do not participate in hybridization: **(A)** Molecular representation of the interaction model between the metformin dication and the electronic delocalization of the aromatic amino acid; **(B)** Geometric optimization for the interaction between metformin and the aromatic amino acids considered: Y, H, F, and W.

To understand the presence of a diffusion path with aromatic amino acids in the interaction pocket, polarity was calculated using the molecule’s hydrophobic and hydrophilic forces of interaction (Supplementary Figure S2). As shown in the magnified image of Figure 1B, there is a pathway led by aromatic functional groups that protruded throughout the helices that intervene in the channel’s translocation diffusion path, which was confirmed with the hydropathy index analysis of the chemical environment of the helices and the helices themselves. This analysis suggests an interaction between the dication and each of the aromatic groups present in the side chain of the alpha helices, as shown in Figure 2A. Energetic and geometric optimization of the system (metformin-aminoacid aromatics), produces a periplanar coupling by non-covalent interactions between the cation and the resonance of the orbitals π of the aromatic ring, as proposed in Figure 2B.

### Metformin-Aromatic Amino Acid Interaction of the Channel Diffusion

After performing the respective couplings considered in the previous figure, these geometric optimization values generated energy values for the system composed of the metformin dication and the aromatic cloud. These energy values are indicated in Table 2. Using the results obtained, the greatest stability, considering the lowest energy values, was obtained for the metformin–tryptophan adducts, followed by metformin–tyrosine and metformin–phenylalanyl and lastly by metformin–histidine.

**TABLE 2:** Interaction energy for deprotonated metformin and the aromatic amino acids.

Chemical system	Interaction energy (hartree)
Metformin–Tyrosine	−1,051.55
Metformin–Tryptophan	−1,107.14
Metformin–Phenylalanyl	−977.10
Metformin–Histidine	−971.24

The following analysis of the results obtained, histidine was the only amino acid that did not present favorable periplanar coupling, which evidences that this interaction is less probable, because, in addition to the acid–base effect, histidine generates a steric impairment that moves it away from periplane. The remaining three aromatic amino acids did present perpendicular interaction with the metformin dication. The calculation of the distance from an imaginary plane demonstrated the lowest coupling distance for metformin–tyrosine at a point in the plane with a value of 3.575 Å and metformin–phenylalanine with the greatest value for a point in the plane with a value of 4.719 Å.

### Electrostatic Potential Map of the Metformin-Aromatic Amino Acid Interaction

**FIGURE 3:**
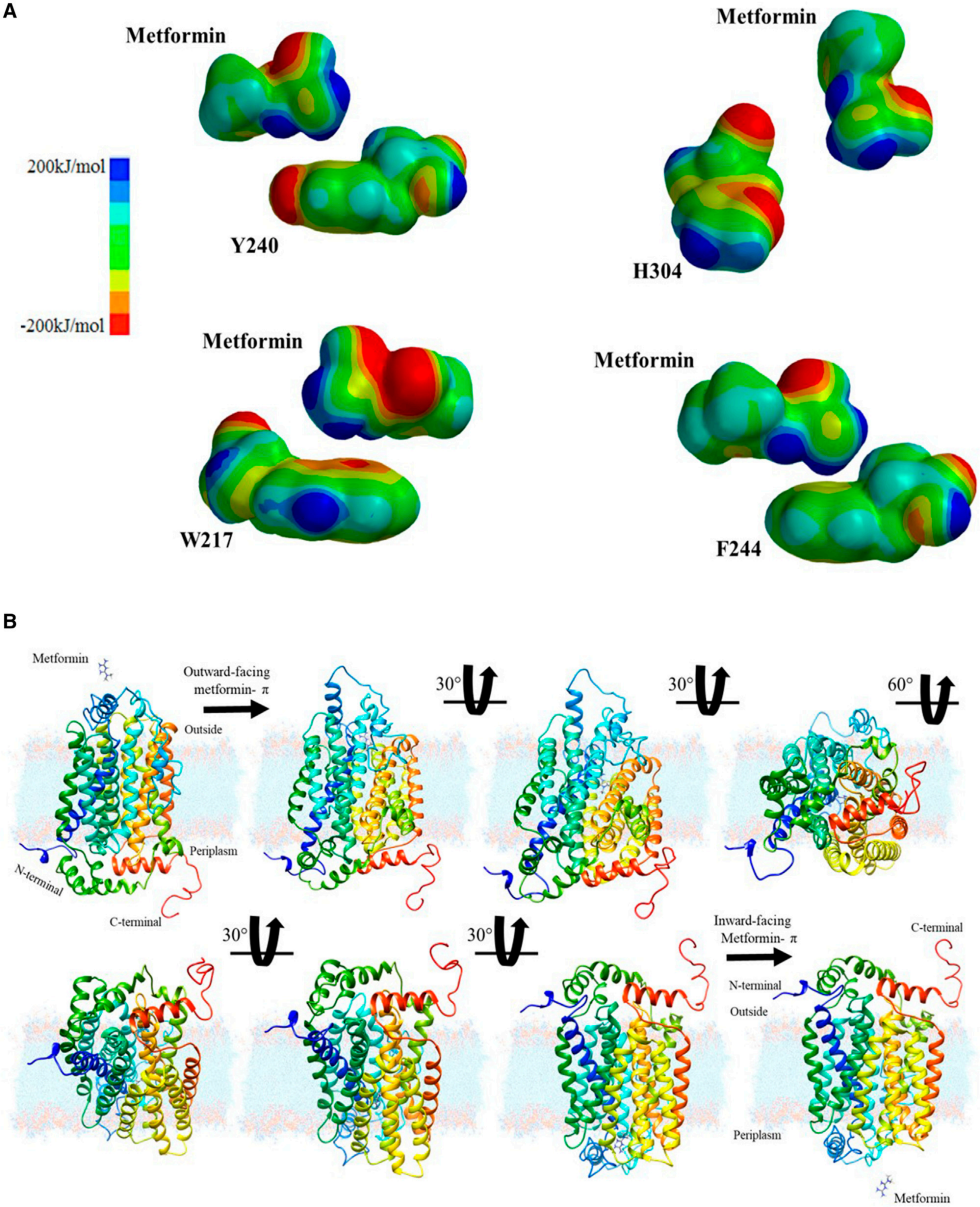
Chemical characterization and proposal for passive diffusion: **(A)** Electrostatic potential surfaces between metformin and aromatic amino acids considered: tyrosine, histidine, phenylalanine, and tryptophan; **(B)** Interaction sequence and translocation movement of metformin in the hOCT1 organic cation channel.

Continuing with the analysis of the interaction between the drug and the aromatic amino acids, additional explanation of a plausible interaction in the diffusion of the drug in the hOCT1 transporter is required. Therefore, a quantum mechanics-based calculation was performed with the Hartree–Föck method to obtain the interaction surfaces of the previously energetically and geometrically optimized structures, all from an electrostatic potential map, to visualize metformin’s anchor points to the transporter, based on the electronic distributions, which may indicate points of greater or lower potential, as shown in Figure 3A.

After obtaining the computational results and metformin’s migration path, it was necessary to determine how the drug is retained and how this interaction favors the conformational change, which leads to the subsequent translocation of the transporter. These cation-aromatic amino acid interactions are mediated by the π-electron cloud, thus facilitating drug absorption by the small intestine. Then, images were created, illustrating the drug transport process, at 30° and 60° from the extracellular region as a dication to the intracellular region in the intestine as shown in Figure 3B, which becomes a proposed mechanism for the passive diffusion and subsequent retention of metformin from a biochemical visualization, for translocation purposes.

A literature search was conducted after the determination of a possible mechanism of interaction and translocation in drug diffusion, to conduct a functional analysis of the mutations associated with the hOCT1 channel and to observe the structural and chemical effects on the transporter’s topology and electronic structure.

### Modeling of the Functional Effects of Mutations in the hOCT1 Organic Cation Channel

To understand the functional effects of similar mutations in the channel, a search was conducted in the databases and literature by selecting non-synonymous, missense mutations, which despite their low frequencies across different populations, affect the pharmacokinetic parameters related to metformin absorption, thus resulting in bioaccumulation in the intestine, liver, and kidneys (Shu et al., 2003; Chen et al., 2010; Zhou et al., 2017; Mato et al., 2018). They were identified in the literature mutations S189L, R206C, and G401S, which are responsible for changing the chemical polarity and topology of the structure (Dakal et al., 2017), altering the function (Shu et al., 2003; Chen et al., 2010; Deanna et al., 2010)

### G401S Mutation Results

**FIGURE 4:**
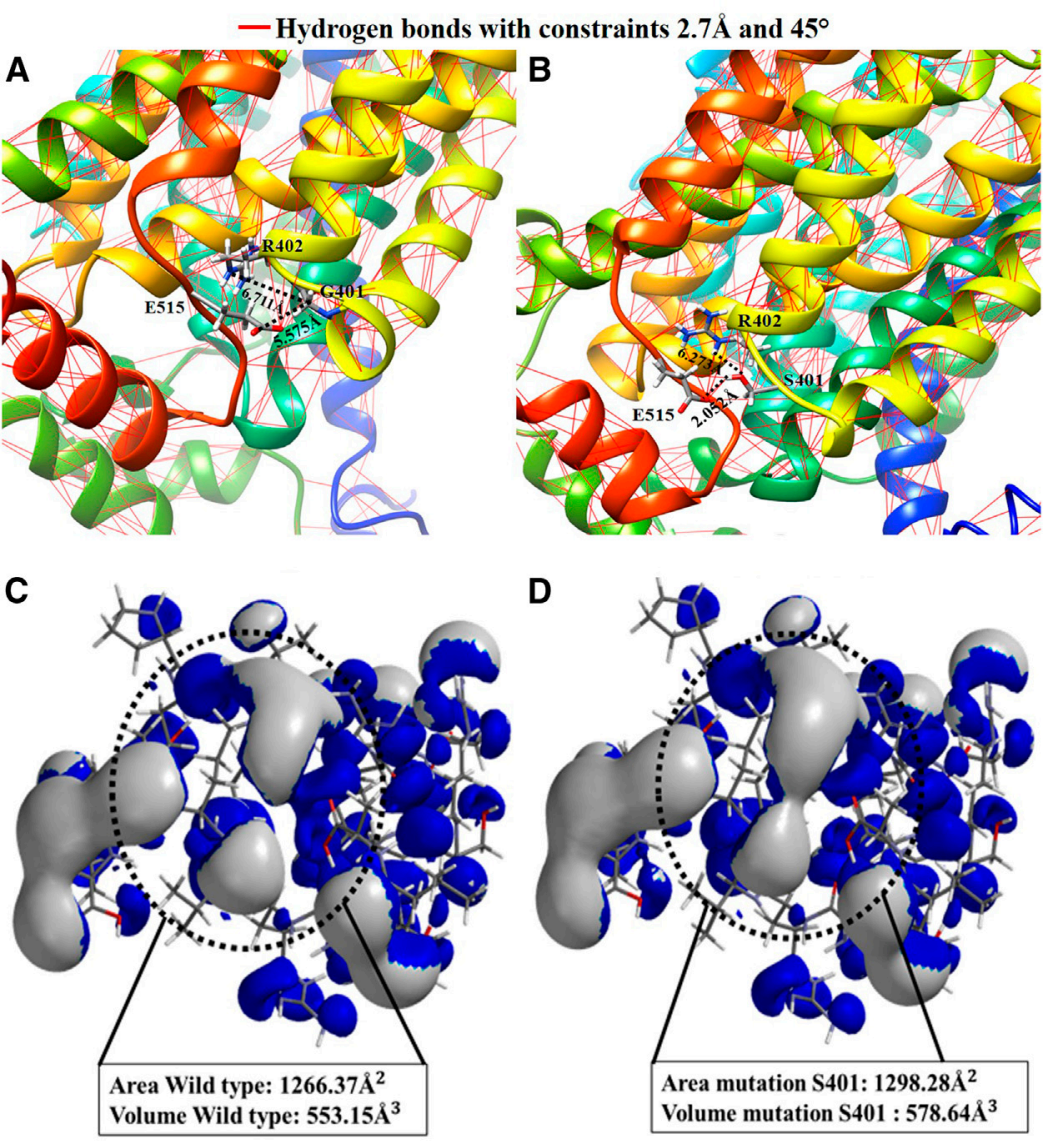
Comparative representation of the effect of the Gly401Ser variant: **(A)** 3D visualization and calculation of the hydrogen bonds in the hOCT1 wild type channel structure; **(B)** 3D visualization and calculation of the hydrogen bonds in the hOCT1 channel mutated by Ser401; **(C)** Ionization potential surface of the hOCT1 wild type channel; **(D)** Ionization potential surface of the hOCT1 channel mutated by Ser401.

The mutated channel and its environment were modeled to compare them to the reported wild type channel modeling (Dakal et al., 2017), to record any changes using computational methods. Figures 4A,B shows the channel’s structural fragment and the ability to generate hydrogen bonds that may alter the structure. With molecular modeling, the measured distances are calculated for both the wild type and the mutation. Figure 4A shows that the amino acid G401 does not have an adequate interaction distance and does not have a functional group to interact. Whereas the G401S mutation (Figure 4B), only one point of interaction with hydrogen bond S401-E515 is observed at a distance of 2.052 Å. In the hydrogen bonding patterns indicated, which are indicated by the red lines, it is observed that S401 is an important anchorage point within the structure. Thus, this specific fragment was selected, aiming to characterize it with an atomic resolution from a quantum chemistry simulation, in order to improve the observable results, as well as the ionization potential surface for both systems. Similarly, the surface areas and volumes were measured based on the surfaces of their electronic structure. The G401S mutation is characterized by the change in polarity it generates because of the alcohol functional group in the side chain of serine, which can create hydrogen bond-based interactions. Accordingly, the hydrogen bonds were calculated throughout the hOCT1 channel for the wild type form and the mutated channel. After assessing the mutation effects with Quantum mechanics calculations, it was possible to observe three new hydrogen bonds corresponding to: 1) S401- R402 interactions from oxygen (–OH), to the hydrogen atom attached to nitrogen (-NH_2_), with a distance of 2.759 Å, 2) S401- E515 interaction of the hydrogen atom (–OH) with the oxygen atom double-bonded to the glutamic amino acid (O=) with a distance of 2.183 Å, and 3) S401- Glu515 interaction because of the bonding of the serine group’s hydrogen atom (–OH) to the oxygen of the carbonyl group (-OH) of glutamic acid, with a distance of 2.453 Å. The distances were calculated for the interaction of the optimized structures using hybrid Quantum Mechanics/Molecular Mechanics (QM/MM), based on the Austin Model1 semi-empirical method, as shown in Supplementary Figure S3. After energy optimization, the ionization potential surface was calculated for the surface area and volume of the wild type channel region in Figure 4C, with values of 1,266.37 Å^2^ and 553.15 Å^3^, respectively. When mutation occurs, the surface area and volume increase considerably to 1,298.28 Å^2^ and 578.64 Å^3^, which could show a change in the electronic structure that may block interaction with metformin, as shown for the surface highlighted in Figure 4D.

### S189L Mutation Results

**FIGURE 5:**
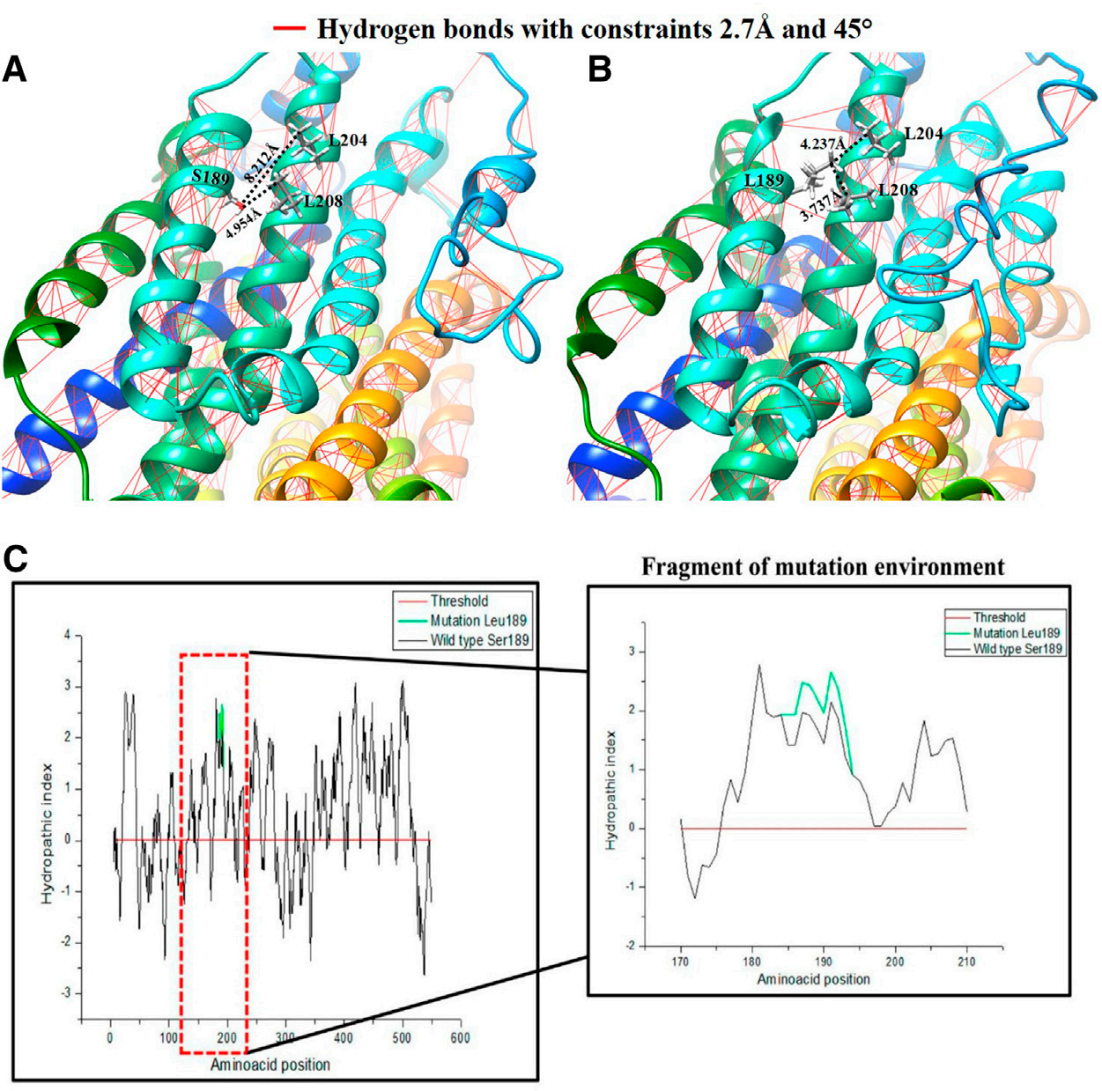
Comparative representation of the effect of the Ser189Leu variant: **(A)** 3D visualization and calculation of hydrogen bonds in the hOCT1 wild type channel structure; **(B)** 3D visualization and calculation of the hydrogen bonds in the hOCT1 channel mutated by Leu189; **(C)** Hydropathy index plot using Kyte and Doolittle coefficients for the hOCT1 wild type and mutated channels; the red box indicates the magnified characteristic around the amino acid at position 189.

For this mutation, modeling of the region in the channel was conducted in the same manner with the mutation position 189, where a considerable polarity change can be observed. The structural change is shown in Figure 5, where a loss of anchor points and hydrogen bonds affecting the structure around position 189 can be observed for a restriction of 2.7 Å, in addition to an approach based on the calculation of the effect on the surrounding polarity from the Kyte and Doolittle coefficients.

As shown in Figures 5A,B, the hOCT1 channel has hydrogen bonds, represented by the red lines. S189 has a hydroxyl group and this functional group allows interactions by the hydrogen bonds with other dipoles when they are separated by a distance of <2.7 Å. When a L189 mutation occurs, this dipole point disappears, for interaction purposes. Moreover, with the quantitative values measured for the vicinity of the mutation in position 189, it can be observed that the mutation generates a closeness between the two α-helixes with a difference for the L204 residue of 3.939 Å and L208 of 1.217 Å, favoring the non-polar interactions between these residues. Figure 5C demonstrates this based on the hydropathy index score with Kyte and Doolittle coefficients for the system. In this analysis, the polarity calculation for the entire hOCT1 channel in composition is presented with the aim of determining the effect of the change on the channel’s chemical environment in the region around position 189 for positive (hydrophobic interactions) or negative (hydrophilic interactions) values, thus showing that mutation increases hydrophobicity when missense, non-synonymous substitution occurs.

In quantitative terms, to assess the topological changes produced to the hOCT1 transporter and their possible association with metformin absorption in the small intestine, the topological calculation represented by the measurement of the angle produced by the three atoms shows that, for the wild type the bonding angle was 112.87° while, for the S189L mutation this was 113.78°. Similarly, after calculating the effects on the dihedral angles involving the two planes generated by the four constituent atoms of the dihedron produced in the side chain of position 189, it was reported that, in the case of the wild type, its value was −78.82°, whereas the value for the mutation’s dihedral planes was −55.06°, as shown in Supplementary Figure S4. The effect of torque and angle favors the interaction between helices because of the increased hydrophobic forces around the mutation position, which may alter the channel’s topology.

### R206C Mutation

**FIGURE 6:**
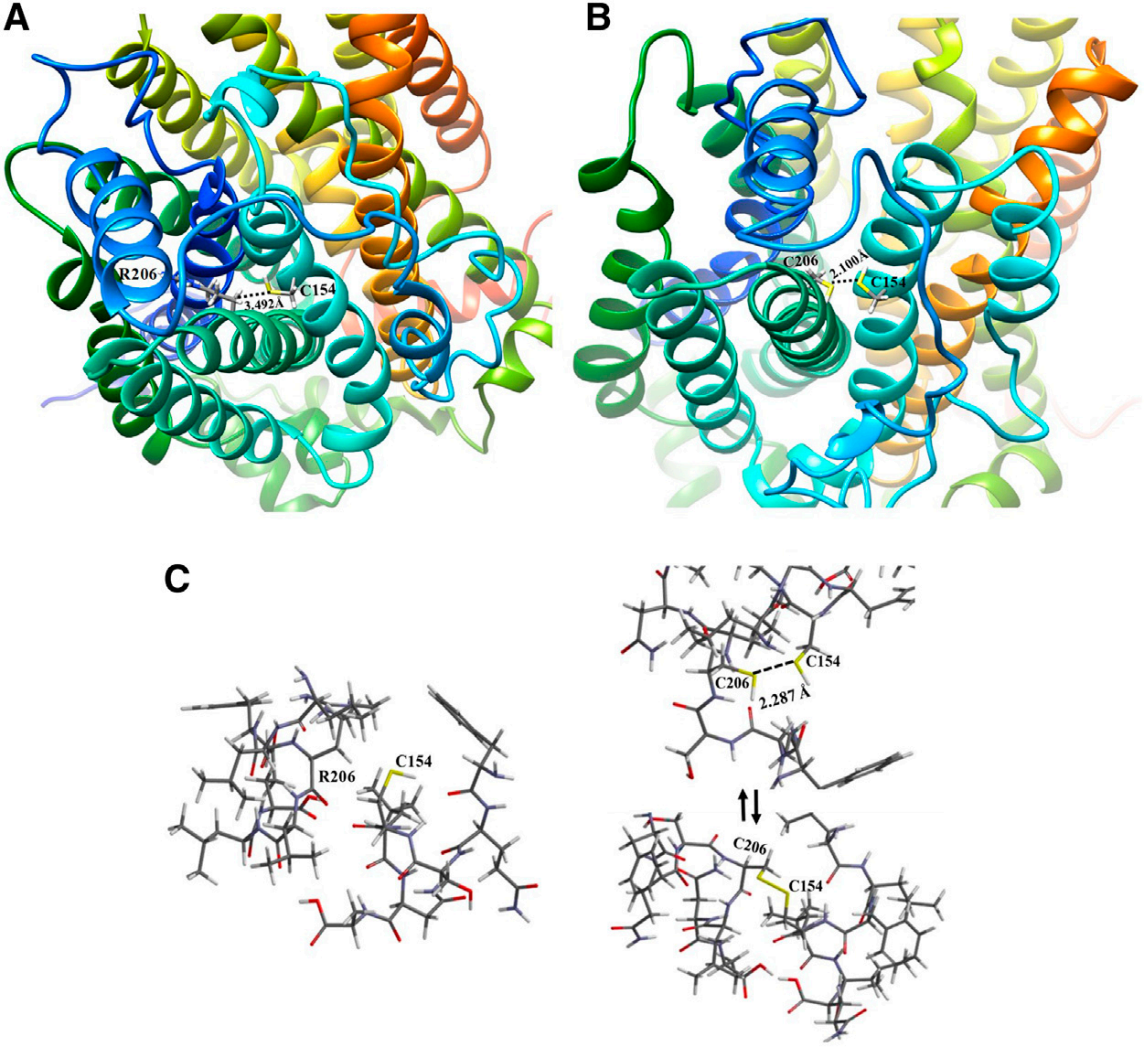
Comparative representation of the effect of the R206C variant: **(A)** 3D visualization of the hOCT1 wild type R206 channel structure; **(B)** 3D visualization of the hOCT1 channel mutated at C206; **(C)** Energy optimization of the neighboring amino acids for R206 and C154 in TM2 and TM3 of the hOCT1 wild type channel (left side); Energy optimization at C206 and C154 with no disulfide bridge between TM2 and TM3 (upper right side); Balanced energy optimization with a closed disulfide bridge for C206 and C154 (bottom right side).

The structural and functional study helped to identify the transmembranes involved in the 3D change and its proximities. For the wild type, R206 is located in transmembrane 3 (TM3), close to transmembrane 2 (TM2), as shown in Figure 6A, which reveals a distance of 3.492 Å from the carbon α of R206 to its neighbor TM2 with the thiol group of the C154 residue, a position that is not reactive as is the carbon of the guanidine group of the arginine aminoacid (R). Then, the R206C mutation was modeled, comparing the potential topological or non-covalent interaction effects with the regions adjacent position 206 and its potential effect on neighboring transmembranes. Figure 6B shows that the mutation occurs at position 206 of TM3, and that there is a cysteine at position 154 in the adjacent TM2, exhibiting a distance of 2.100 Å between the two thiol groups of the amino acid cysteines, which may create a disulfide bridge that may cause a 3D change that would alter the channel’s topology, thus affecting its interaction with metformin.

Considering the model generated from the mutation at position 206 and the possibility of forming a disulfide bridge, the energy optimization study was conducted through hybrid simulation QM/MM for both the wild type and mutation with the formation of a disulfide bridge and open chain structures, thus observing whether the range is suitable for interaction when the bonding distance is measured, as shown in Figure 6C. The calculation for the energy value associated with the R206-C154 wild type transporter was −3,689.488 kJ/mol; moreover, for the mutation, the bonding distance was first measured between sulfur atoms, thus generating a value of 2.287 Å, a distance that is considered sufficient for forming the disulfide bridge. Similarly, the optimization energies of the channel region with no R206-C154 disulfide bond (no disulfide bridge), and the formation of an R206-C154 disulfide bond (with disulfide bridge) were measured with values of −3,765.068 and −3,700.327 kJ/mol, respectively. It is clearly evident that these values are much lower than those for wild type structures and consider a lower potential number. This process benefits from enthalpy because a more stable configuration is achieved once a mutation occurs in the structures; however, it leads to a topological alteration.

### Assessment of the Frequency of SLC22A1 Mutations in Diabetic Patients

Because of the functional relevance of these mutations in metformin pharmacokinetics, the presence of G401S (rs34130495), S189L (rs34104736), and R206C (616C > T) on the SLC22A1 gene was evaluated in a sample of diabetic patients. This analysis demonstrated that these variants are monomorphic in the Colombian population sample under study.

## Discussion

Metformin is one of the drugs most extensively used worldwide for treating (T2DM) (Markowicz-Piasecka et al., 2016; Bailey, 2017; Sanchez-Rangel and Inzucchi, 2017). The studies on the supply of metformin demonstrate that it enters the body encapsulated in a magnesium stearate-based excipient. As soon as it reaches the intestine, the stearate is hydrolyzed and absorbed, similar to Mg such that metformin is released and with the small intestines’ main microspecies being the dication form. Because this species is positively charged, the cation channel is responsible for transporting organic molecules. To propose the diffusion model for metformin according to the values calculated from the hydropathy index, the aromatic chemical functions of the side chain move toward the path formed between transmembrane helices TMH2, TMH4, TMH5 and TMH7, as shown in Figure 1A. When considering the aromatic functional groups directed by the hydrophobic pathway, an interaction between the cation and the aromatic π system must be considered. The electronic mobility causes both orbital phases to have a high electronic distribution and negative partial charge; however, the other orbitals that form the bond have an electronic deficiency such that aromatic compounds have a quadrupole effect. After analysis of the geometrically optimized metformin dication and the amino acids capable of delocalizing electron density, only tyrosine, phenylalanine, and tryptophan had this type of periplanar interaction because histidine does not interact in that manner with the drug, as shown in the results. This assessment helped explain how metformin undergoes protonation at physiological pH in the small intestine, thus forming the dication. When the drug approaches histidine within the organic cation channel, the intermolecular proton is transferred because the double-bonded nitrogen has a higher s-orbital nature. Because of its proximity to the nucleus, it has the most localized load and easier protonation, which has a direct impact on aromaticity because there is no interaction between the π system and the cation. This phenomenon explains why histidine does not accept the interaction between the dication and the p orbitals in its calculation. Similarly, this is demonstrated by the calculation of the alpha fractions; because the pH in the small intestine is 4.90; moreover, the percentage of histidine microspecies, i.e., protonated species, in the sp^2^ nitrogen was 98.01. Therefore, its protonation is inevitable and does not generate the interaction for diffusion. The hOCT1 organic cation channel is 554 amino acids in length; this channel may be subject to changes associated with the low absorption of metformin, which gives rise to a response related to low drug concentration in the intestine.

The first mutation to discuss is the change at position 401 from glycine to serine. From the chemical perspective, this mutation shows a change in polarity because the side chain goes from having two hydrogens to having an alcohol functional group, therefore increasing the channel’s polarity and gaining a potential anchor point for hydrogen-bond type interactions. To better understand the electronic structure change, an analysis was conducted based on a quantum-mechanical approach, which helped to obtain ionization potential surface. Furthermore, after modeling of the mutation, an increase could be observed in the surface area and electronic volume associated around the mutation at 401. This may result in a new point of interaction with metformin, thus preventing its translocation in the small intestine. Similarly, from a molecular point of view, metformin enters the channel as a dication; however, because it mutates into an amino acid with an alcohol functional group, three new dipole points for hydrogen bond interaction develop, which are capable of interacting with the hydroxyl group dipole and forming an adduct with metformin in the said ion-dipole position, thus preventing its diffusion into the system and affecting translocation of the drug.

This mutation occurs at position 189, where a change in polarity can be noted; i.e., serine, with an alcohol functional group in its side chain, turns into leucine, an amino acid with isobutyl hydrocarbon group. A loss of points for hydrogen bond interaction can be observed in the structural modeling, because of the serine hydroxyl, which leads us to propose an approach to understand the variant from a nonpolar perspective. The hydrophobic interactions favor this change, and tend to move toward the alpha helices or, when unavailable, toward the membrane. This mutated system promotes non-polar interactions such as the London dispersion force, which occurs because of the proximity of electron densities that produce attraction. Therefore, a change in the angle of the alpha carbon and the dihedral planes of the peptide bond can be observed when implementing a hybrid QM-MM system. Consequently, the helix becomes deformed and seeks better interaction with the membrane and the adjacent helix. Moreover, the Kyte and Doolittle coefficients assign positive score values to the alpha helix structure environment, thus favoring hydrophobic characteristics. The mutation marked in green in the graph confirms the change in polarity because the values of >0, which is the threshold, are related to a hydrophobic environment.

The changes taking place in the angles are significant enough to lead to structural consequences that favor hydrophobic interactions. When the two adjacent transmembranes (TM2 and TM4) approach each other, the diffusion path is altered with regard to its interaction with metformin, which would affect absorption. Note that, when hydrophobic interactions are enhanced, the organic cation channel favors interaction with the membrane, therefore affecting translocation of the drug.

Analysis of the system shows that a change in angle may favor L189’s search for hydrophobic interactions, as shown in Supplementary Figures S4A,B. When mutation takes place, a 0.91° change in the angle can be seen at the alpha carbon; this change causes the alpha helix to bend, as shown in the belts of Figure 5A, above the mutation, bringing transmembrane 4 and transmembrane 2 cloS, which is important for metformin’s diffusion path and its subsequent translocation. Similarly, a change is observed in the dihedral angle of the wild type with respect to the dihedral angle of the mutated channel, decreasing by around −23.76°, and makes it possible to quantify the effect of mutation at the quantum mechanical level (Supplementary Figures S4C,D).

This mutation takes place at position 206 of transmembrane3, where arginine, a polar and basic amino acid, is substituted by cysteine, a polar but small amino acid. The effect on the environment is not very significant, but because of its location, it may favor the formation of a disulfide bridge because the adjacent transmembrane, transmembrane2, has a cysteine at position 154. After better assessment of the structure from the image, it was possible to observe that the proximity of the cysteine amino acids in transmembrane2 and transmembrane3 is visible at a distance of 2.287 Å; therefore, the formation of a disulfide bridge can be expected, which is a very probable and common type of interaction to take place in proteins in general, for cysteine amino acids with a sulfur-like thiol functional group on their side chain. After energy optimization and analysis of the values determined in the results, very close energy data were reported for both structures at the energy optimization level. This indicates that the formation of a disulfide bridge is plausible, which would bring transmembranes 2 and 3 cloS to each other, moving away from the diffusion and stabilization path under the dication-aromatic π system to metformin, which works against translocation.

Finally, although the modeled mutations are monomorphic in the CLM (Colombian) population of 1,000 genomes, data nonetheless, given that our sample composed exclusively of diabetic patients with several years of diagnosis and pharmacological treatment with different degrees of response to medications, we expected to detect their presence in some of them. However, this was not the case because the results obtained indicate that G401S (rs341a30495), S189L (rs34104736) and R206C (616C > T) are monomorphic among this study group. Although these mutations have been mostly reported across European populations (Maruthur et al., 2014; Dujic et al., 2015) and, to a lesser extent, Asian populations (Chen et al., 2010). we were interested, in exploring their existence/presence in the Colombian population. Our analyses showed the absence of these mutations in our sample despite being composed exclusively of Colombian diabetic patients. The inability to detect these mutations in our sample might be an indicative of allelic heterogeneity due to evolutionary and demographic differences among populations. This result suggests that there must be other variants in our population that modify the response to the drug; therefore, additional studies on metformin pharmacogenetics are required to identify genetic variants involved in the response to the drug for this population.

## Conclusion

In this study, both structural and functional visualization and conceptualization allowed us to understand and propose a plausible diffusion and translocation model for Organic Cation Transporter hOCT1 based on a theoretical analysis of the interaction of the metformin dication with the aromatic residues of the diffusion path’s side chain. These amino acids were selected according to their aromatic nature because they were identified along the path followed by metformin and its interaction through the cation-π electronic cloud. This interaction may be important in the translocation that the hOCT1 channel undergoes to transport the drug, inducing a conformational change, facilitating the entry of the drug into the target tissues. One limitation of this study is the sample size, which may explain that could not be observed mutations in this population, due to their low frequency. Further research is highly recommended to identify mutations involved in the response to metformin in the colombian population.

## Data Availability

The raw data supporting the conclusions of this article will be made available by the authors, without undue reservation.

## References

[B1] Abramson J. Smirnova I. Kasho V. Verner G. Kaback H. R. Iwata S. (2003). Structure and mechanism of the lactose permease of *Escherichia coli* . Science 301 (5633), 610–615. 10.1126/science.1088196 12893935

[B2] Artimo P. Jonnalagedda M. Arnold K. Baratin D. Csardi G. de Castro E. (2012). ExPASy: SIB bioinformatics resource portal. Nucleic Acids Res., 40, W597. 10.1093/nar/gks400 22661580 PMC3394269

[B3] Atlassian Confluence 6.9.0. (2007). Chemicalize-chemaxon ltda. Available at: https://chemaxon.com/products/chemicalize (Accessed August 11, 2018)

[B4] Bailey C. J. (2017). Metformin: historical overview. Diabetologia 60 (9), 1566–1576. 10.1007/s00125-017-4318-z 28776081

[B5] Cardona S. M. Kim S. V. Church K. A. Torres V. O. Cleary I. A. Mendiola A. S. (2018). Role of the fractalkine receptor in CNS autoimmune inflammation: new approach utilizing a mouse model expressing the human CX3CR1I249/m280 variant. Front. Cell. Neurosci. 12, 1–17. 10.3389/fncel.2018.00365 30386211 PMC6199958

[B6] Cardona-Pemberthy V. Rendón M. Beltrán J. C. Soto-Ospina A. Muñoz-Gomez A. Araque-Marín P. (2018). Genetic variants, structural, and functional changes of Myelin Protein Zero and Mannose-Binding Lectin 2 protein involved in immune response and its allelic transmission in families of patients with leprosy in Colombia. Infect. Genet. Evol. 61, 215–223. 10.1016/j.meegid.2018.04.002 29627640

[B7] Caro M. A. (2018). Evaluación de una base común en la etiología genética de obesidad, diabetes tipo 2, hipertensión y dislipidemia, en una población producto de mezcla genética. Medellín, Colombia: Universidad de Antioquia.

[B8] Chen E. C. Khuri N. Liang X. Stecula A. Chien H. C. Yee S. W. (2017). Discovery of competitive and noncompetitive ligands of the organic cation transporter 1 (OCT1; SLC22A1). J. Med. Chem. 60 (7), 2685–2696. 10.1021/acs.jmedchem.6b01317 28230985

[B9] Chen L. Takizawa M. Chen E. Schlessinger A. Segenthelar J. Choi J. H. (2010). Genetic polymorphisms in organic cation transporter 1 (OCT1) in Chinese and Japanese populations exhibit altered function. J. Pharmacol. Exp. Therapeut. 335 (1), 42–50. 10.1124/jpet.110.170159 PMC2957788 20639304

[B10] Ciarimboli G. Gautron S. E. S. (2015). Organic cation transporters: integration of physiology, pathology, and pharmacology. Cham. Switzerland: Springer Nature Switzerland AG.

[B11] Dakal T. C. Kumar R. Ramotar D. (2017). Structural modeling of human organic cation transporters. Comput. Biol. Chem. 68, 153–163. 10.1016/j.compbiolchem.2017.03.007 28343125

[B51] Deanna L. K. Yee S. W. Giacomini K. M. (2010). The Pharmacogenomics of Membrane Transporters Project: Research at the interface of genomics and transporter pharmacology. Clin. Pharmacol. Ther. 87 (1), 109–116. 10.1038/clpt.2009.226 19940846 PMC2923224

[B12] Dujic T. Zhou K. Donnelly L. A. Tavendale R. Palmer C. N. Pearson E. R. (2015). Association of organic cation transporter 1 with intolerance to metformin in type 2 diabetes: a GoDARTS study. Diabetes 64 (5), 1786–1793. 10.2337/db14-1388 25510240 PMC4452716

[B13] Duque C. (2011). Efeccto de la mezcla genetica en Diabetes Mellitus Tipo 2, enpoblación Antioqueña. Medellín, Colombia: Universidad de Antioquia.

[B14] Florez J. C. (2017). The pharmacogenetics of metformin. Diabetologia 60 (9), 1648–1655. 10.1007/s00125-017-4335-y 28770331 PMC5709222

[B15] Foretz M. Guigas B. (2019). Understanding the glucoregulatory mechanisms of metformin in type 2 diabetes mellitus. Nat. Rev. Endocrinol. 15 (10). 569–589. 10.1038/s41574-019-0242-2 31439934

[B16] Gasteiger E. Hoogland C. Gattiker A. Duvaud S. Wilkins M. R. Appel R. D. (2005). The proteomics protocols handbook-protein identification and analysis tools on the ExPASy server. Totowa, NJ: Humana Press.

[B17] Green L. A. (2015). Investigating the impact of OCT transporter genotype on metformin- induced vitamin B12 deficiency. PhD thesis. Liverpool (United Kingdom): University of Liverpool.

[B18] Guex N. Peitsch M. C. (1997). SWISS-MODEL and the Swiss-PdbViewer: an environment for comparative protein modeling. Electrophoresis 18 (15), 2714–2723. 10.1002/elps.1150181505 9504803

[B19] Kroetz D. L. Yee S. W. Giacomini K. M. (2010). The pharmacogenomics of membrane transporters project: research at the interface of genomics and transporter pharmacology. Clin. Pharmacol. Ther. 87 (1), 109–116. 10.1038/clpt.2009.226 19940846 PMC2923224

[B20] Liang X. Giacomini K. M. (2017). Transporters involved in metformin pharmacokinetics and treatment response. J. Pharmacol. Sci. 106 (9), 2245–2250. 10.1016/j.xphs.2017.04.078 28495567

[B21] Lin L. Yee S. W. Kim R. B. Giacomini K. M. (2015). SLC transporters as therapeutic targets: emerging opportunities. Nat. Rev. Drug Discov. 14 (8), 543–560. 10.1038/nrd4626 26111766 PMC4698371

[B22] Mahrooz A. Parsanasab H. Hashemi-Soteh M. B. Kashi Z. Bahar A. Alizadeh A. (2015). The role of clinical response to metformin in patients newly diagnosed with type 2 diabetes: a monotherapy study. Clin. Exp. Med. 15 (2), 159–165. 10.1007/s10238-014-0283-8 24740684

[B23] Markowicz-Piasecka M. Huttunen K. M. Mateusiak L. Mikiciuk-Olasik E. Sikora J. (2016). Is metformin a perfect drug? Updates in pharmacokinetics and pharmacodynamics. Curr. Pharmaceut. Des. 23 (17), 2532–2550. 10.2174/1381612822666161201152941 27908266

[B24] Maruthur N. M. Gribble M. O. Bennett W. L. Bolen S. Wilson L. M. Balakrishnan P. (2014). The pharmacogenetics of Type 2 Diabetes: a systematic review. Diabetes Care 37 (3), 876–886. 10.2337/dc13-1276 24558078 PMC3931386

[B50] Mato P. E. M. Guewo-Fokeng M. Faadiel Essop M. Mark P. Owira O. (2018). Genetic polymorphisms of organic cation transporter 1 (OCT1) and responses to metformin therapy in individuals with type 2 diabetes A systematic review. Syst. Rev. Meta-Analysis Med. 97, e11349. 10.1097/MD.0000000000011349

[B25] Nies A. T. Hofmann U. Resch C. Schaeffeler E. Rius M. Schwab M. (2011). Proton pump inhibitors inhibit metformin uptake by organic cation transporters (OCTs). PLoS One 6 (7), e22163–11. 10.1371/journal.pone.0022163 21779389 PMC3136501

[B26] Parra M. V. (2012). Genética de la Resistencia a la Insulina y Diabetes Mellitus 2 en Población Antioqueña. Medellín, Colombia: Universidad de Antioquia. available at: https://revistas.udea.edu.co/index.php/iatreia/article/view/8329.

[B27] Pascale E. Mato M. Guewo-Fokeng M. Faadiel Essop M. Mark P. Owira O. (2018). Genetic polymorphisms of organic cation transporter 1 (OCT1) and responses to metformin therapy in individuals with type 2 diabetes. A systematic review Systematic Review and Meta-Analysis Medicine® OPEN 1. 0(June). 10.1097/MD.0000000000011349

[B28] Pettersen E. F. Goddard T. D. Huang C. C. Couch G. S. Greenblatt D. M. Meng E. C. (2004). UCSF Chimera--a visualization system for exploratory research and analysis. J. Comput. Chem. 25 (13), 1605–1612. 10.1002/jcc.20084 15264254

[B29] Quality T. (2007). Center on education policy, 2007. Challenges 5, 1–11. 10.1038/nprot.2010.5.I-TASSER

[B30] Sanchez-Rangel E. Inzucchi S. E. (2017). Metformin: clinical use in type 2 diabetes. Diabetologia 60 (9), 1586–1593. 10.1007/s00125-017-4336-x 28770321

[B31] Santoro A. B. Botton M. R. Struchiner C. J. Suarez-Kurtz G. (2018). Influence of pharmacogenetic polymorphisms and demographic variables on metformin pharmacokinetics in an admixed Brazilian cohort. Br. J. Clin. Pharmacol. 84 (5), 987–996. 10.1111/bcp.13522 29352482 PMC5903234

[B32] Schlessinger A. Khuri N. Giacomini K. M. Sali A. (2013). Molecular modeling and ligand docking for solute carrier (SLC) transporters. Curr. Top. Med. Chem. 13 (7), 843–856. 10.2174/1568026611313070007 23578028 PMC4056341

[B33] Shu Y. Brown C. Castro R. A. Shi R. J. Lin E. T. Owen R. P. (2008). Effect of genetic variation in the organic cation transporter 1, OCT1, on metformin pharmacokinetics. Clin. Pharmacol. Ther. 83 (2), 273–280. 10.1038/sj.clpt.6100275 17609683 PMC2976713

[B34] Shu Y. Leabman M. K. Feng B. Mangravite L. M. Huang C. C. Stryke D. (2003). Evolutionary conservation predicts function of variants of the human organic cation transporter, OCT1, Proc. Natl. Acad. Sci. U.S.A., 100 (10), 5902–5907. 10.1073/pnas.0730858100 12719534 PMC156299

[B35] Shu Y. Sheardown S. A. Brown C. Owen R. P. Zhang S. Castro R. A. (2007). Effect of genetic variation in the organic cation transporter 1 (OCT1) on metformin action. J. Clin. Invest. 117 (5), 1422–1431. 10.1172/JCI30558DS1 17476361 PMC1857259

[B36] Song I. S. Shin H. J. Shim E. J. Jung I. S. Kim W. Y. Shon J. H. (2008). Genetic variants of the organic cation transporter 2 influence the disposition of metformin. Clin. Pharmacol. Ther. 84 (5), 559–562. 10.1038/clpt.2008.61 18401339

[B37] Takane H. Shikata E. Otsubo K. Higuchi S. Ieiri I. (2008). Polymorphism in human organic cation transporters and metformin action, Pharmacogenomics 9, 415–422. 10.2217/14622416.9.4.415 18384255

[B38] Ubarretxena-Belandia I. Baldwin J. M. Schuldiner S. Tate C. G. (2003). Three-dimensional structure of the bacterial multidrug transporter EmrE shows it is an asymmetric homodimer. EMBO J. 22 (23), 6175–6181. 10.1093/emboj/cdg611 14633977 PMC291852

[B39] Wang D. Jonker J. W. Kato Y. Kusuhara H. Schinkel A. H. Sugiyama Y. 2002). Involvement of organic cation transporter 1 in hepatic and intestinal distribution of metformin, J. Pharmacol. Exp. Therapeut. 302(2), 510–515. 10.1124/jpet.102.034140.Metformin 12130709

[B40] Wavefunction (1991). Spartan 18’. Wavefunction, Available at: https://www.wavefun.com/corporate/more_spartan.html.

[B41] Yang J. Yan R. Roy A. Xu D. Poisson J. Zhang Y. (2015). The I-TASSER Suite: protein structure and function prediction. Nat. Meth. 12 (1), 7–8. 10.1038/nmeth.3213 PMC4428668 25549265

[B42] Yang P. Nicolás J. C. Galván C. A. Vélez P. Ronco L. Da. Díaz G. T. (2014b). Efectividad de la metformina en pacientes con diabetes tipo II según variantes en el gen SLC22A1. Acta Bioquímica Clínica Latinoamericana 48(2),229–235.

[B43] Yoon H. Cho H. Y. Yoo H. D. Kim S. M. Lee Y. B. (2013). Influences of organic cation transporter polymorphisms on the population pharmacokinetics of metformin in healthy subjects. AAPS J. 15 (2), 571–580. 10.1208/s12248-013-9460-z 23417334 PMC3675749

[B44] Zhang X. Shirahatti N. V. Mahadevan D. Wright S. H. (2005). A conserved glutamate residue in transmembrane helix 10 influences substrate specificity of rabbit OCT2 (SLC22A2). J. Biol. Chem. 280 (41), 34813–34822. 10.1074/jbc.M506342200 16087669

[B45] Zhang Y. (2008). I-TASSER server for protein 3D structure prediction. BMC Bioinf. 9, 40. 10.1186/1471-2105-9-40 PMC2245901 18215316

[B46] Zhou F. Zhu L. Wang K. Murray M. (2017). Recent advance in the pharmacogenomics of human Solute Carrier Transporters (SLCs) in drug disposition, Adv. Drug Deliv. Rev. 116, 21–36. 10.1016/j.addr.2016.06.004 27320645

