# Supplementary material for: Diffusion Mechanism Modeling of Metformin in Human Organic Cationic Amino Acid Transporter one and Functional Impact of S189L, R206C, and G401S Mutation
Source: Front Pharmacol. 2021 Feb 9;11:587590. doi: 10.3389/fphar.2020.587590 (PMC7917475; doi:10.3389/fphar.2020.587590)
Supplement: Supplementary file 1 [file DataSheet1.pdf]

Supporting material

Diffusion Mechanism Modeling of metformin in Human Organic Cationic Amino Acid Transporter one and Functional Impact of S189L, R206C, and G401S Mutation.

Leydy Cano[1], Alejandro Soto-Ospina[1,2], Pedronel Araque[2,5], Maria Antonieta Caro-Gomez[1], Maria Victoria Parra-Marin[3], Gabriel Bedoya[1] and Constanza Duque[4*]

1. Universidad de Antioquia, Medellín, Colombia.

2. Universidad EIA, Envigado, Colombia.

3. Tecnológico de Antioquia, Medellín, Colombia.

4. Universidad Cooperativa de Colombia, Medellín Colombia.

5. Cecoltec, Medellín, Colombia

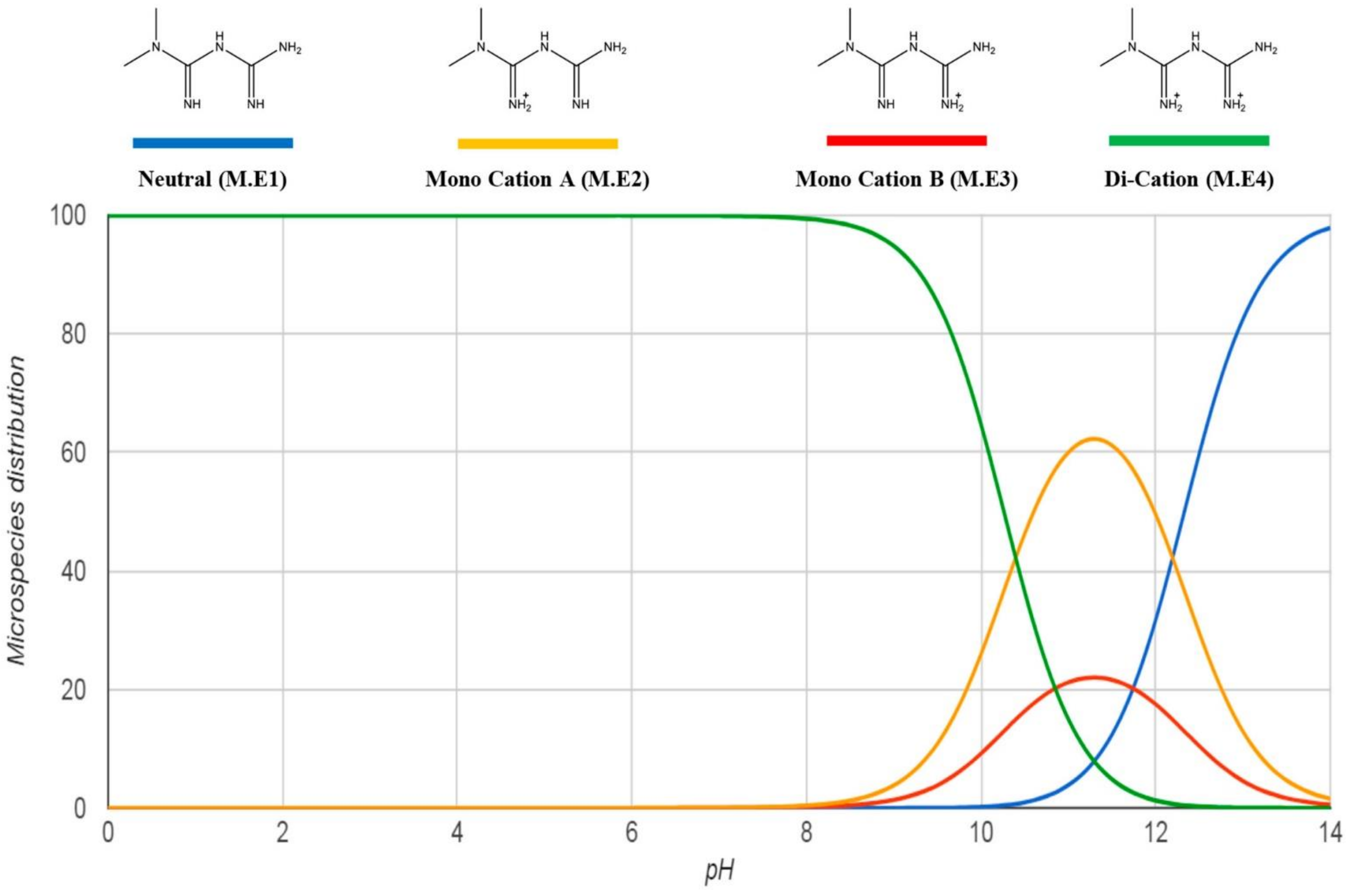

Fig. S1 *Metformin characterization for calculation of the α (alpha) fractions*

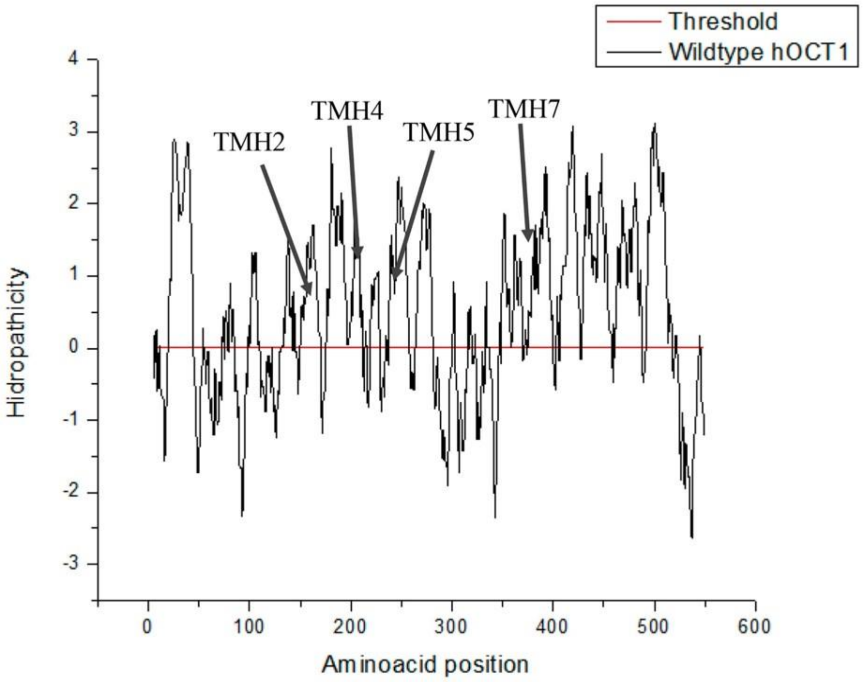

A

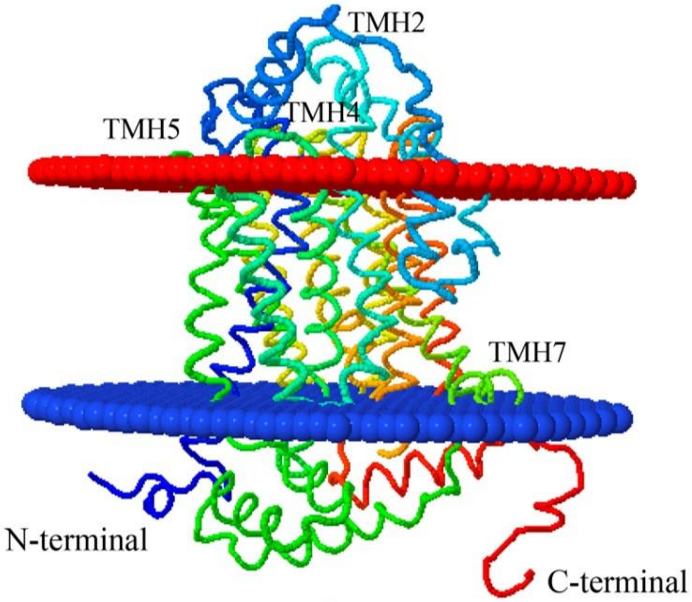

B

Fig. S2 *Characterization of Human Organic Cation Transporter hOCT1: A) Hydropathy index plot for hOCT1; B) Structure of transmembrane α-helices involved in the passive diffusion of the metformin cation*

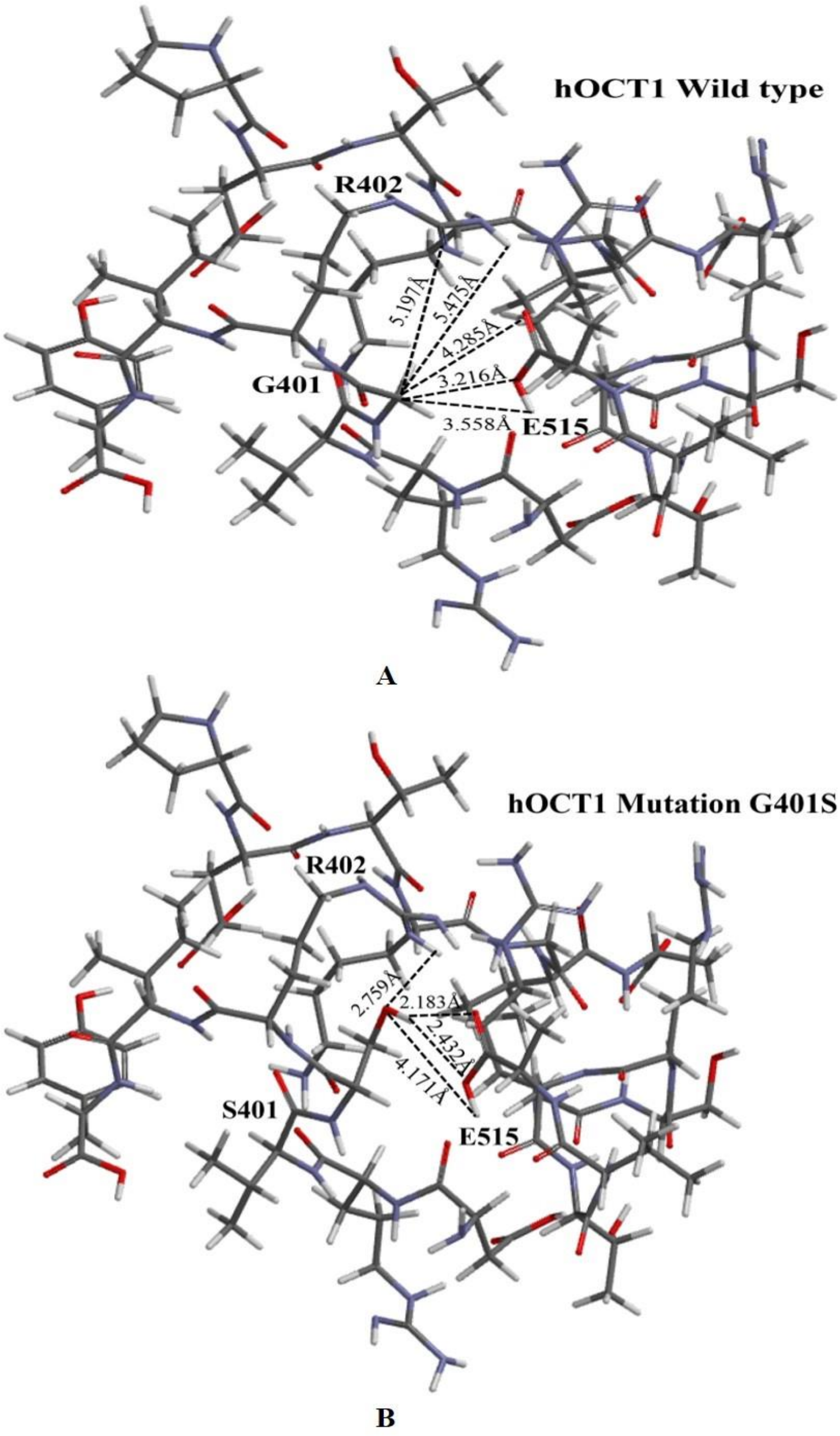

Fig.S3. *Stick representation of amino acid structure in the proximity of position 401 in hOCT1: A) Bonding distances for the 401Gly structure; B) Bonding distances for the Ser401 structure*

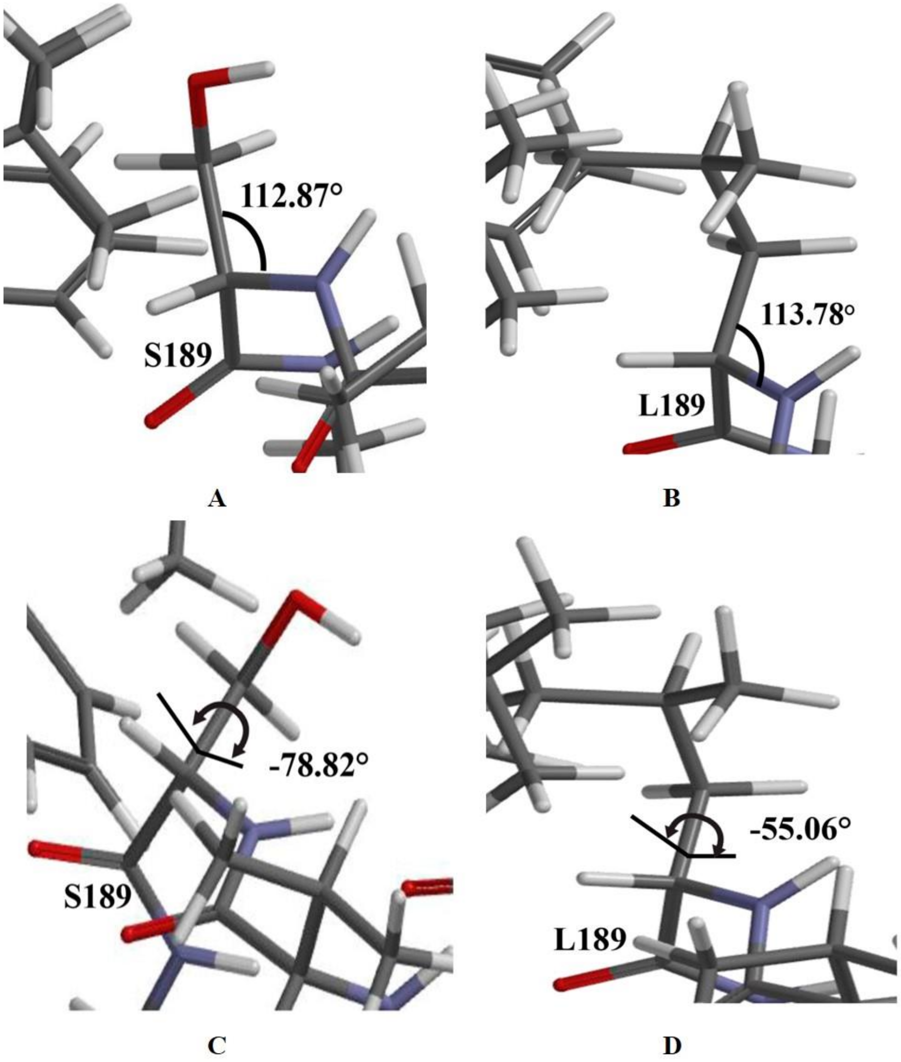

Fig.S4. *Structural comparison of the region adjacent to position 189 of the hOCT1 channel: A) 3D visualization of the change in angle of the alpha carbon in the wild type Ser189 channel; B) 3D visualization of the change in angle of the alpha carbon in the wild type Leu189 mutated channel; C) 3D visualization of the change in the dihedral angle between alpha carbon planes in the wild type Ser189 channel; D) 3D visualization of the change in the dihedral angle of the alpha carbon planes Leu189 mutated channel*
